# Responsible remembering and forgetting as contributors to memory for important information

**DOI:** 10.3758/s13421-021-01139-4

**Published:** 2021-01-20

**Authors:** Dillon H. Murphy, Alan D. Castel

**Affiliations:** Department of Psychology, University of California Los Angeles, Los Angeles, Los Angeles, CA 90095 USA

**Keywords:** Directed forgetting, Responsible remembering, Schematic support, Selective rehearsal

## Abstract

The ability to control both what we remember and what is forgotten can enhance memory. The present study used an item-method directed forgetting paradigm to investigate whether participants strategically remembered items they were responsible for remembering rather than items a hypothetical friend was responsible for remembering. Specifically, participants were presented with a 20-word list (either unrelated words or items to pack for a camping trip) with each word followed by a cue indicating whether the participant (You) or their “friend” (Friend) was responsible for remembering the word. When asked to recall all of the words, regardless of the cue, recall was sensitive to the You and Friend instructions such that participants demonstrated elevated recall for the items they were responsible for remembering, and participants also strategically organized retrieval by recalling You items before Friend items. Additionally, when asked to judge the importance of remembering each item, participants’ recall and recognition were sensitive to item importance regardless of cue. Taken together, the present experiments revealed that the strategic encoding of important information and the forgetting of less important, goal-irrelevant information can maximize memory utility and minimize negative consequences for forgetting. Thus, we provide evidence for a metacognitive process we are calling *responsible forgetting*, where people attempt to forget less consequential information and focus on remembering what is most important.

## Introduction

When we forget important things that we expected to remember, this poor metacognitive outcome has obvious negative consequences like disappointing exam scores or social embarrassment after forgetting someone’s name. In instances when we are presented with more information than can be remembered, we frequently exhibit strategic control, the ability to focus on and direct resources towards valuable information (e.g., Ariel, Dunlosky, & Bailey, 2009; Castel, Benjamin, Craik, & Watkins, 2002; Castel, McGillivray, & Friedman, 2012). How we prioritize memory for important information, or information with negative consequences if forgotten, is a notion we termed *responsible remembering* (see Murphy & Castel, 2020).

Although not as obviously useful as remembering important information, forgetting is also a critical component of memory. Specifically, to avoid forgetting important things, people may have developed the ability to forget information they do not need to remember so that they can focus on important information. For example, remembering where you parked your car yesterday or the day before is not very helpful for finding your car today. Similarly, remembering old phone numbers and addresses may interfere with the recall of current ones. Thus, there may be a functional quality of forgetting such that people strategically remember important information and forget outdated or unimportant information to reduce competition for target information (cf. Anderson, Bjork, & Bjork, 1994; Bjork, Bjork, & Anderson, 1998; Fawcett & Hulbert, 2020). As a result, situations in which forgetting serves an implicit or explicit personal need exemplify the need for *responsible forgetting*.

When presented with to-be-remembered information, a person’s habitual response is often to attempt to remember as much information as possible. However, in some cases, it can be beneficial to forget less important information to facilitate memory for critical information and engage in responsible remembering. Cognitive control refers to the ability to engage in functional, goal-directed behavior that allows one to overcome previously learned habitual behaviors to accommodate competing task demands (Chiew & Braver, 2017; Diamond, 2013; Egner, 2017; Miller & Cohen, 2001). Applied to responsible remembering, cognitive control may be a critical component for engaging in responsible forgetting.

Unlike many memory tasks, directed forgetting tasks present participants with to-be-remembered as well as to-be-forgotten words (item-method directed forgetting; see Bjork & Bjork, 1996 for list method). Largely pioneered by Bjork, LaBerge, and Legrand (1968), directed forgetting tasks present participants with items one at a time, and after each item, a cue indicates whether participants should remember or forget the item (see also Woodward & Bjork, 1971). Similarly, some directed forgetting tasks present words paired with either positive or negative point values that count towards participants’ scores on the task if they later recall the word (see Castel, Farb, & Craik, 2007). Because recalling the words with negative point values would reduce their score, participants should have no motivation to remember words associated with negative values and only remember the words resulting in gains if recalled.

Although often deemed an undesirable memory failure, forgetting can lead to memory benefits such that compared with controls, recall for information not expected to be tested (or paired with negative values) tends to be poor (the costs of forgetting), while recall for information expected to be tested (or positively valued) tends to be enhanced, exemplifying the benefits of forgetting (e.g., Bjork & Bjork, 1996; Friedman & Castel, 2011; for reviews, see Basden & Basden, 1998; Bjork, 1998; MacLeod, 1998). Thus, responsibly forgetting information, perhaps unimportant or outdated information, may enhance the recall of target information and lead to responsible remembering.

After a free recall test where participants recall words they had been instructed to remember (and not those that were paired with a cue to forget the word), directed forgetting tasks often include a surprise recognition test. Specifically, participants are presented with the to-be-remembered and to-be-forgotten words as well as words that were not presented (lures) and are asked to indicate whether each word is “new” (was not presented in the study phase) or “old” (was presented in the study phase). If participants had indeed forgotten the to-be-forgotten words, they would indicate that the to-be-forgotten words were new. However, participants sometimes later recognize words that they had been instructed to forget, indicating some memory for these words (e.g., Thompson, Fawcett, & Taylor, 2011; Zacks, Radvansky, & Hasher, 1996).

This measure of forgetting in directed forgetting tasks can serve as an indicator of inhibitory control and exemplifies the *retrieval inhibition theory* (Basden & Basden, 1998; Basden, Basden, & Gargano, 1993; Bjork, 1989; Geiselman & Bagheri, 1985; Geiselman, Bjork, & Fishman, 1983; Weiner & Reed, 1969; see also Verde, 2012; see Racsmány & Conway, 2006, for episodic inhibition account; see Sahakyan & Kelley, 2002, for the context-change account), the theory that the inhibition of to-be-forgotten items facilitates retrieval of to-be-remembered items. Alternatively, researchers have also argued for selective rehearsal accounts of directed forgetting (Bjork, 1972; MacLeod, 1975; Sheard & MacLeod, 2005; Tan, Ensor, Hockley, Harrison, & Wilson, 2020), such that participants strategically rehearse and encode to-be-remembered items rather than to-be-forgotten items. Specifically, selective rehearsal accounts posit that presented items are maintained in working memory until participants are cued to remember or forget the item, and to-be-remembered items are elaboratively rehearsed (to transfer to long-term memory) while rehearsal for to-be-forgotten items is stopped. Thus, functional forgetting due to inhibition or selective rehearsal may be used to strategically enhance memory.

Regardless of the mechanism behind directed forgetting, forgetting may be a critical aspect of a functional memory system such that forgetting items that do not need to be remembered may facilitate memory for items that do need to be remembered. Exemplifying this effect, previous work has demonstrated that offloading to-be-remembered information (e.g., saving information to a computer, writing things down) facilitates memory for other to-be-remembered information by reducing the extent to which the offloaded information interferes with target information (Risko & Dunn, 2015; Risko & Gilbert, 2016; Sparrow, Liu, & Wegner, 2011; Storm & Stone, 2014). Thus, when remembering a list of items, selectively rehearsing goal-relevant items or inhibiting less important information may facilitate the retrieval of valuable items or items with negative consequences if forgotten.

When presented with information to remember (or forget), general representations or heuristics influence how we perceive and remember the world and impact how we value information (see McGillivray & Castel, 2017). This use of prior knowledge can enhance memory (a form of “schematic support,” as described by Craik & Bosman, 1992) such that knowledge in a domain can facilitate memory for other information in that domain. Thus, schematic support may be beneficial for remembering important information; however, little research has investigated the role of schematic support and item importance in directed forgetting tasks.

## The Current Study

Previous work has modified memory through explicit instruction to forget (directed forgetting tasks). However, in the current study, a cue indicated whether the participant or a hypothetical friend was responsible for remembering each item (as opposed to a transactive memory system, see Hollingshead, 2001 and Wegner, 1986; or collaborative memory, see Rajaram & Pereira-Pasarin, 2010) on a list of to-be-remembered words (unassociated words or items to pack for a camping trip). Specifically, we presented participants with a number of items (20) greater than the typical memory span of an individual (Cowan, 2001; Unsworth & Engle, 2007) to determine how participants allocate cognitive recourses to maximize memory utility. We expected participants to engage in responsible forgetting by selectively rehearsing and remembering goal-relevant information and forgetting goal-irrelevant information from the list. Thus, the current paradigm could result in responsible forgetting such that participants prioritize their cognitive resources for items on the list that they are responsible for remembering.

We were also interested in whether responsible forgetting occurs in situations offering schematic support. Specifically, while we expected participants to show enhanced recall for the items they were responsible for remembering, if participants use schematic support to enhance their memory, then they may be able to remember goal-relevant, important items to pack for a camping trip (see McGillivray & Castel, 2017) even if their friend was responsible for remembering them. Thus, we expected responsible remembering to be enhanced in conditions offering schematic support such that items of greatest importance, or biggest consequences if forgotten, are best recalled.

To further investigate memory for items that participants were responsible for remembering compared with items that their friend was responsible for remembering, we had participants complete a surprise cue-pairing test (Experiment 1) where they were presented with all words from the study phase and asked to indicate whether they or their friend were responsible for remembering each item. Additionally, to investigate whether certain items were more or less effectively encoded, we had participants complete a surprise recognition test (Experiments 2 and 3) where they were presented with words from the study phase as well as not-studied words and had to indicate whether the items had been presented. Similar to the recall test, in both the cue-pairing and recognition tests, we expected participants to demonstrate enhanced accuracy for items they were responsible for remembering.

## Experiment 1

In Experiment 1, participants were presented with a list of words, with each word followed by a cue indicating whether the participant (“You”) or a hypothetical friend (“Friend”) was responsible for remembering the word. Participants were then given a free recall test for all of the words, regardless of the cue, as well as a surprise cue-pairing test. While some participants were presented with a list of unrelated words, others were presented with a list of words offering schematic support (items to pack for a camping trip, adapted from McGillivray & Castel, 2017) to make remembering and forgetting more salient and to introduce motivation to remember or forget certain words. We expected participants to prioritize and best remember items they were responsible for remembering compared with items their friend was supposed to remember, regardless of the type of words (unassociated or schematic support). However, we expected recall to be sensitive to item importance, regardless of the cue, when benefiting from schematic support. Additionally, in line with selective rehearsal accounts of directed forgetting, we expected participants to demonstrate better cue-pairing accuracy for items they were responsible for remembering compared with items their friend was responsible for remembering, and that this would not differ based on the type of stimuli.

## Method

### Participants

Participants were 60 undergraduate students (age: *M* = 20.52 years, *SD* = 1.64) recruited from the University of California Los Angeles Human Subjects Pool and received course credit for their participation. A sensitivity analysis using G*Power (Faul, Erdfelder, Lang, & Buchner, 2007) indicated that for a repeated-measures, between-subjects ANOVA with two groups (stimulus type: schematic support, unassociated) and two measurements (cue: Friend, You), with a low correlation between repeated measures (recall for Friend and You items, *r* = -.16), assuming alpha = .05, power = .80, the smallest effect size the design could reliably detect is η2 = .05. Participants were tested individually or in groups of up to eight individuals in a laboratory session lasting approximately 1 h.

### Materials and procedure

Participants were informed that they would be presented with a list of words that they and a (hypothetical) friend needed to remember and that after each word was presented, a cue would indicate whether they (You) or their friend (Friend) was responsible for remembering the word. Participants were randomly assigned to either be presented with unassociated words (*n* = 30) or asked to imagine that they and a friend were going camping (items offering schematic support; *n* = 30). If presented with items to remember for a camping trip, participants were told that they would be presented with a list of items that they and their friend needed to remember to bring on the trip (see Appendix Table 1 for stimuli adapted from McGillivray & Castel, 2017).

For each participant, half of the words were randomly designated as to-be-remembered words for the participants and half were designated as words their friend was responsible for remembering. Each word was preceded by a 1-s fixation cross, then appeared on the screen, one at a time, in random order, for 3 s followed by the cue for an additional 2 s. After the presentation of all 20 words, participants were given a 1-min free-recall test in which they were asked to recall all of the words that both they and their friend needed to remember from the just-presented list.

Following the recall test, participants completed a surprise cue-pairing test where they were shown the words from the just-presented list (in random order) and asked to indicate whether they or their friend were responsible for remembering the word. Participants also provided confidence judgments on a scale from 0 to 100 (with 0 being not at all confident and 100 being very confident) and were given as much time as they needed for this portion of the task. Finally, participants reported what encoding strategies (if any) they had used for the items they were responsible for remembering and which strategies (if any) they had used for the items their friend was responsible for remembering. Specifically, participants indicated whether they simply read each word as it appeared, repeated the words as much as possible, used sentences to link the words together, developed mental images of the words, grouped the words in a meaningful way, or utilized some other strategy (participants could select “some,” “all,” or “none”).

Lastly, to evaluate the general importance of each item on the list of items to bring on a camping trip, we recruited a separate sample of undergraduate students (*n* = 60) from the University of California Los Angeles Human Subjects Pool. These participants were shown all 20 items and then asked to rate each item on a scale from 0 (not important) to 100 (very important). See Appendix Table 2 for mean importance ratings (see also McGillivray & Castel, 2017) and Appendix C for more information.

## Results

The results are divided into six primary sections: recall, output order, importance, cue-pairing performance, confidence on the cue-pairing test, and encoding strategy use. In each section, we investigated differences as a function of cue (Friend, You) and stimulus type (words offering schematic support, unassociated words). To reinforce each effect, we computed the Bayes Factor (BF; a ratio of the marginal likelihood of the null model and a model suggesting group differences). The data are reported as either in favor of the null hypothesis (which would be supported by a large BF_01_) or the alternative hypothesis (which would be supported by a large BF_10_; see Kass & Raftery, 1995, for more information).

### Recall

**Fig. 1:**
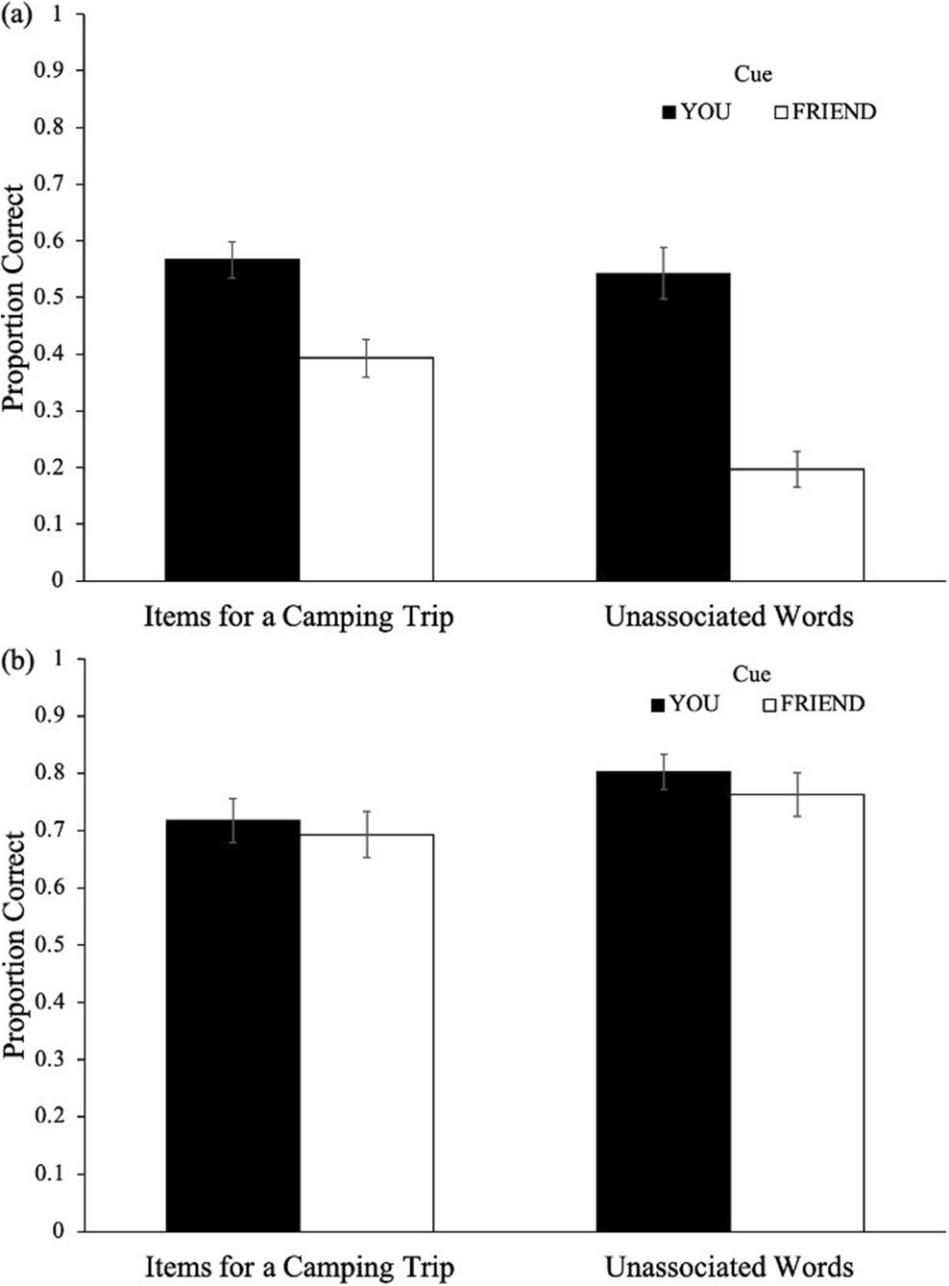
Recall performance **a** and cue-pairing test performance **b** as a function of cue and stimulus type in Experiment 1. Error bars reflect the standard error of the mean

Recall performance as a function of cue and stimulus type is shown in Fig. 1a. A 2 (cue: Friend, You) × 2 (stimulus type: schematic support, unassociated) repeated-measures ANOVA on recall performance revealed a main effect of cue [*F*(1, 58) = 43.46, *p* < .001, η2 = .41, BF_10_ > 100], such that participants recalled more You items (*M* = .56, *SD* = .21) than Friend items (*M* = .30, *SD* = .20). Additionally, results revealed a main effect of stimulus type [*F*(1, 58) = 11.81, *p* = .001, η2 = .17, BF_10_ > 100], such that participants presented with items offering schematic support (*M* = .48, *SD* = .12) recalled a greater proportion of items than participants presented with unassociated words (*M* = .37, *SD* = .13). Additionally, cue interacted with condition [*F*(1, 58) = 4.83, *p* = .032, η2 = .05, BF_10_ > 100].

To investigate differences in recall between You items for each stimulus type, an independent-samples *t*-test was conducted, but Levene’s test of equality of variances indicated a violation of the equal variance assumption (*p* = .026). Welch’s *t*-test did not reveal differences in recall of You items between the schematic support (*M* = .57, *SD* = .17) and unassociated word conditions (*M* = .54, *SD* = .25), [*t*(51.72) = .42, *p* = .675, *d* = .11, BF_01_ = 3.54]; however, participants recalling words offering schematic support recalled more Friend items (*M* = .39, *SD* = .19) than those recalling unassociated words (*M* = .20, *SD* = .17), [*t*(58) = 4.29, *p* < .001, *d* = 1.11, BF_10_ > 100]. Thus, participants demonstrated similar recall of You items across conditions but elevated recall of Friend items in the schematic-support condition.

### Output order

To examine how participants organized retrieval, a Gamma correlation between the output position of each correct item (with larger numbers indicating later output) and the corresponding cue (You coded as 1, Friend coded as 0) was computed across participants. A strong positive correlation would indicate that participants recalled Friend items before You items while a negative correlation would indicate recalling You items before Friend items. A correlation near 0 would indicate no organization of recall according to cue. Results revealed that, overall, participants recalled You items before Friend items (γ = -.25, *p* < .001). Additionally, we computed Gamma correlations at the participant level in each condition (schematic support: *M* = -.15, *SD* = .50; unassociated words: *M* = -.38, *SD* = .66), but an independent-samples *t*-test did not reveal group differences in the organization of recall [*t*(50) = 1.46, *p* = .151, *d* = .41, BF_01_ = 1.51].

### Importance

**Fig. 2:**
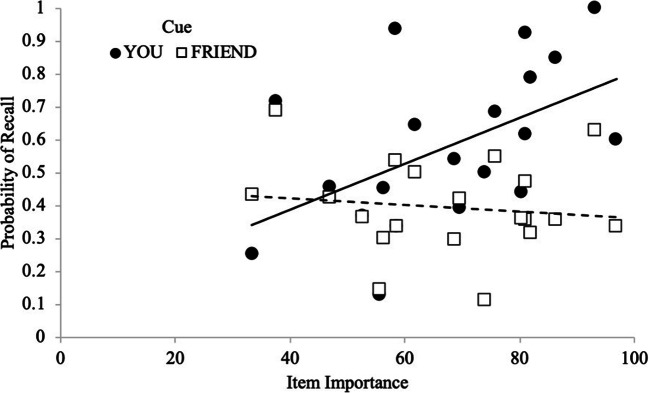
Probability of recall as a function of cue and item importance with regression lines in Experiment 1

In the schematic-support condition, to determine if participants prioritized recall for important items, a Gamma correlation between recall accuracy and item importance was computed across participants. A strong positive correlation would indicate that participants prioritized recall for important items while a negative correlation would indicate that participants prioritized recall of unimportant items. A correlation near 0 would indicate no sensitivity to importance. Results revealed that, overall, participants’ recall was sensitive to importance (γ = .14, *p* = .006), indicating that participants recalled important items better than less important items. We also computed Gamma correlations between recall accuracy and item importance at the participant level for each cue (You and Friend items), and these correlations (You: *M* = .37, *SD* = .44; Friend: *M* = -.01, *SD* = .50) served as the dependent variable in a paired-samples *t*-test. Results revealed that participants were more sensitive to importance (see Fig. 2) for the items they were responsible for remembering compared with items their friend was responsible for remembering [*t*(26) = 3.74, *p* < .001, *d* = .72, BF_10_ = 37.03].

### Cue-pairing test

Results of the cue-pairing test as a function of cue and stimulus type are shown in Fig. 1b. To examine differences in performance (scored as proportion correct) on the cue-pairing test,^1^ a 2 (cue: Friend, You) × 2 (stimulus type: schematic support, unassociated) repeated-measures ANOVA did not reveal a main effect of stimulus type [*F*(1, 58) = 3.01, *p* = .088, η2 = .05, BF_10_ = 1.02], such that participants presented with words offering schematic support (*M* = .71, *SD* = .18) paired a similar proportion of words with the correct cue to participants presented with unassociated words (*M* = .78, *SD* = .17). Additionally, results did not reveal a main effect of cue [*F*(1, 58) = 1.57, *p* = .216, η2 = .03, BF_01_ = 2.54], such that participants correctly paired a similar proportion of You items (*M* = .76, *SD* = .19) as Friend items (*M* = .73, *SD* = .21). Moreover, cue did not interact with condition [*F*(1, 58) = .11, *p* = .743, η2 < .01, BF_01_ = 9.69]. Thus, participants’ associative memory for items was not sensitive to cue, suggesting that participants had generally accurate associative memory for who was responsible for remembering each item.

To investigate whether cue-pairing accuracy was sensitive to item importance, a Gamma correlation was computed across participants. Results revealed that, overall, participants’ cue-pairing accuracy was not sensitive to importance (γ = -.04, *p* = .505). Additionally, we computed Gamma correlations at the participant level for each cue, and these correlations (You: *M* = .08, *SD* = .40; Friend: *M* = .00, *SD* = .54) served as the dependent variable in a paired-samples *t*-test. Results revealed that participants were similarly insensitive to importance for the items they were responsible for remembering compared with items their friend was responsible for remembering [*t*(21) = .82, *p* = .420, *d* = .18, BF_01_ = 3.31].

### Confidence

To determine if participants’ confidence on the cue-pairing test differed as a function of cue and stimulus type, a 2 (cue: Friend, You) × 2 (stimulus type: schematic support, unassociated) repeated-measures ANOVA did not reveal a main effect of stimulus type [*F*(1, 58) < .01, *p* = .992, η2 < .01, BF_01_ = 2.64], such that participants presented with words offering schematic support (*M* = 75.85, *SD* = 17.56) were similarly confident as participants presented with unassociated words (*M* = 75.81, *SD* = 16.42). However, results revealed a main effect of cue [*F*(1, 58) = 15.96, *p* < .001, η2 = .22, BF_10_ > 100], such that participants were more confident in You items (*M* = 79.26, *SD* = 19.10) than Friend items (*M* = 72.40, *SD* = 17.03), but cue did not interact with condition [*F*(1, 58) = .03, *p* = .856, η2 < .01, BF_10_ = 13.68].

### Strategy use

Prior research has suggested that effective encoding strategies lead to better memory performance and include interactive imagery, sentence generation, and grouping, whereas less effective strategies involve passive reading and simple repetition (Hertzog, McGuire, & Lineweaver, 1998; Richardson, 1998; Unsworth, 2016). In the present study, we coded self-reported encoding strategies in terms of their level of effectiveness and differentiated less effective strategies and strategies that support deeper levels of processing. Specifically, to examine variation in self-reported encoding strategy use between You and Friend items, we computed an effective strategies variable that was the proportion of effective strategies reported as used by participants (i.e., using sentences to link the words together, developing mental images of the words, and grouping the words in a meaningful way). A 2 (cue: Friend, You) × 2 (stimulus type: schematic support, unassociated) repeated-measures ANOVA on effective strategy use did not reveal a main effect of stimulus type [*F*(1, 58) = 1.24, *p* = .271, η2 = .02, BF_01_ = 2.22]. However, results revealed a main effect of cue [*F*(1, 58) = 19.78, *p* < .001, η2 = .25, BF_10_ > 100], such that participants reported using more effective strategies for You items (*M* = .33, *SD* = 32) than Friend items (*M* = .16, *SD* = .27), potentially supporting selective rehearsal accounts of directed forgetting. Moreover, cue did not interact with condition [*F*(1, 58) = 2.49, *p* = .120, η2 = .03, BF_10_ > 100].

## Discussion

In Experiment 1, we examined how participants prioritized memory for items they and a friend were responsible for remembering. Additionally, we investigated how the prioritization of memory was influenced by schematic support and item importance. Similar to previous work on directed forgetting (e.g., Bjork & Bjork, 1996; MacLeod, 1998), recall for information not expected to be tested (Friend items) was poorer than recall for information expected to be tested (You items), exemplifying the benefits of forgetting. Additionally, items offering schematic support were recalled better than unassociated words, such that participants recalled a similar number of You items in each condition but fewer Friend items when the words were unassociated, consistent with schema-related encoding (e.g., Bransford & Johnson, 1972; Mandler, 1984), such that pre-existing knowledge can enhance memory for to-be-remembered information in this domain.

Moreover, the enhanced recall of camping trip items compared with unassociated words is consistent with the congruity effect (Craik & Tulving, 1975), whereby memory is enhanced when the context (going on a camping trip) forms an integrated unit with the words presented (i.e., tent, matches, water, etc.). Furthermore, individual-item processing (the encoding of item-specific information) and relational processing (the encoding of similarities among a category; Einstein & Hunt, 1980; Hunt & McDaniel, 1993) may have aided memory performance via the encoding of both types of information (see Hunt & Einstein, 1981). Thus, participants appear to have allocated their cognitive resources in favor of items they were responsible for remembering but recalled additional items they were not responsible for remembering as a result of the benefits of schematic support (cf., Castel, 2005; Craik, 2002; Craik & Bosman, 1992; Golding, Long, & MacLeod, 1994; McGillivray & Castel, 2017).

Although there were no differences in performance between the groups or cues (and participants were not sensitive to importance) on the cue-pairing test, when participants were responsible for remembering an item for the camping trip (You), their recall was sensitive to importance. Specifically, participants better remembered important items compared with unimportant items that they were responsible for remembering while showing no sensitivity for importance for items their friend was responsible for remembering. These findings suggest that both You and Friend items were still accessible in memory (they had generally accurate associative memory for who was responsible for remembering each item) and that participants may have inhibited Friend items to engage in responsible forgetting to enhance memory of goal-relevant, important information, potentially fitting inhibition accounts of directed forgetting (see also Aguirre, Gómez-Ariza, Andrés, Mazzoni, & Bajo, 2017).

## Experiment 2

In Experiment 1, participants recalled You items better than Friend items and after the recall test for all items regardless of the cue, participants completed a cue-pairing test to determine whether their cue-item associative memory was intact. Results did not reveal differences in associative memory as a function of cue and cue-pairing accuracy was not sensitive to importance (and this was replicated with no preceding recall test^2^). In Experiment 2, we further investigated the effects of the You and Friend cues on recall performance and sensitivity to importance by adding a control condition where words were presented without the You and Friend cues. Additionally, we replaced the cue-pairing test with a recognition test to determine whether participants had better recognition memory for items they were responsible for remembering compared with items their friend was responsible for remembering, similar to the recall pattern observed in Experiment 1. Participants also provided their own importance ratings for each item at the end of the task to determine if recall and recognition are sensitive to participant-level importance ratings and if participants’ sensitivity to importance depends on the You and Friend cues. While we expected recall to be sensitive to cue and importance as observed in Experiment 1, we also expected participants to demonstrate better recognition accuracy for items they were responsible for remembering.

## Method

### Participants

Participants (after exclusions: *n* = 62, age: *M* = 23.69 years, *SD* = 2.09) were recruited from Amazon’s Mechanical Turk, a Web site that allows users to complete small tasks for pay. Participants received $1.50 for completing the experiment, which took approximately 10 min. All participants were required to have completed a high school degree in the USA to participate. Participants were excluded from analysis if they admitted to cheating (e.g., writing down answers) on a post-task questionnaire (they were told they would still receive credit if they cheated). This exclusion process resulted in two exclusions. A sensitivity analysis indicated that for a two-group (cues, no cues) test of independent means (recall and recognition performance), assuming alpha = .05, power = .80, for a two-tailed test, the smallest effect size the design could reliably detect is *d* = .72.

### Materials and procedure

Participants were randomly assigned to one of two conditions: items paired with cues as in Experiment 1 (*n* = 30) or items not accompanied by a cue (*n* = 32). The procedure and materials were similar to Experiment 1, however, we only presented participants with items offering schematic support. Additionally, in the no-cue condition, participants were only told to imagine that they were going camping and that they would be presented with a list of items that they needed to remember (there were no instructions involving a hypothetical friend). In the cued condition, each item was preceded by a 1-s fixation cross, then appeared on the screen, one at a time, for 3 s followed by the cue for an additional 2 s (as in Experiment 1). However, in the no-cue condition, each item was preceded by a 3-s fixation cross, then appeared on the screen, one at a time, for 3 s (thus the study phase was the same duration in both conditions).

After the study and recall phases, rather than a surprise cue-pairing test, participants completed a surprise recognition test whereby participants were shown the items from the just-presented list as well as 20 lures (in random order) and asked to indicate whether each item was on the list of presented items to bring for the camping trip. Participants also provided confidence judgments as in Experiment 1. After completing the recognition test, participants were asked to rate the importance of each item on a scale from 0 to 100 (with 0 being not important and 100 being very important). Lastly, participants reported their use of encoding strategies as in Experiment 1.

## Results

### Recall

To examine differences in the proportion of items recalled between conditions, an independent-samples *t*-test was conducted but Levene’s test of equality of variances indicated a violation of the equal variance assumption (*p* = .005). Welch’s *t*-test revealed similar recall whether there were cues (*M* = .42, *SD* = .14) or no cues (*M* = .48, *SD* = .24), [*t*(50.04) = 1.34, *p* = .187, *d* = .34, BF_01_ = 1.87]. However, to investigate recall performance as a function of cue in the cued condition, a paired-samples *t*-test revealed that the proportion of You items recalled (*M* = .52, *SD* = .20) was greater than the proportion of Friend items recalled (*M* = .32, *SD* = .19), [*t*(29) = 4.02, *p* < .001, *d* = .73, BF_10_ = 77.77]. Thus, recall for information expected to be tested (You items) was better than recall for information not expected to be tested (Friend items), exemplifying the benefits of forgetting and replicating Experiment 1.

### Output order

We also investigated whether participants prioritized the retrieval of items according to who was responsible for remembering them. A Gamma correlation between the output position of each correct item and the corresponding cue (You coded as 1, Friend coded as 0) was computed across participants. Results revealed that, overall, participants recalled You items before Friend items (γ = -.21, *p* = .013), similar to Experiment 1.

### Importance

To determine if participants prioritized recall for important items, a Gamma correlation between recall accuracy and item importance was computed across participants. Results revealed that, overall, participants’ recall was sensitive to importance (γ = .20, *p* < .001), indicating that participants recalled items rated as important better than items receiving lower importance ratings. However, an independent-samples *t*-test did not reveal group differences in sensitivity to importance (cue: *M* = .17, *SD* = .39; no cue: *M* = .20, *SD* = .46), [*t*(55) = .31, *p* = .758, *d* = .08, BF_01_ = 3.58].

**Fig. 3:**
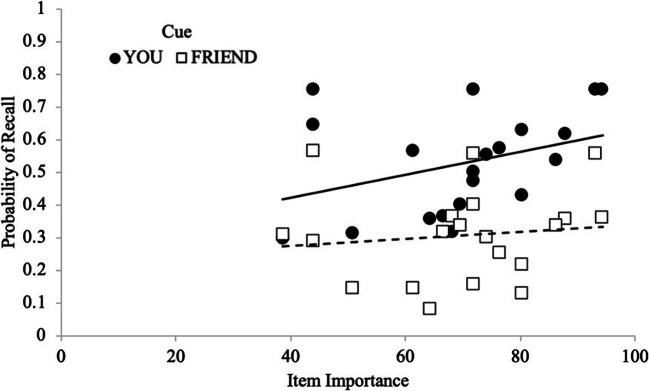
Probability of recall as a function of cue and item importance with regression lines in Experiment 2

At the participant level, Gamma correlations for each cue (You: *M* = .10, *SD* = .57; Friend: *M* = .27, *SD* = .57) served as the dependent variable in a paired-samples *t*-test. Contrary to Experiment 1, results did not reveal cue-related differences in sensitivity to importance [*t*(27) = .99, *p* = .333, *d* = .19, BF_10_ = 3.21], such that participants’ item level importance ratings^3^ did not significantly relate to recall probability as a function of cue. Furthermore, as seen in Fig. 3, when considering each item’s probability of recall as a function of its average importance rating, participants were similarly sensitive to importance ratings for You items (You: *M* = .21, *SD* = .44) and Friend items (*M* = .10, *SD* = .45), [*t*(27) = 1.46, *p* = .155, *d* = .28, BF_01_ = 1.92]. Thus, participants prioritized remembering items they were responsible for remembering and were sensitive the importance of the items but were not differentially sensitive to item importance based on who was responsible for remembering them.

### Recognition

To determine whether there were differences in participants’ ability to distinguish between studied and novel items, *A’* was calculated for each participant using hit rates (i.e., correct identifications of presented items; *M* = .85, *SD* = .15) and false-alarm rates (i.e., instances in which participants incorrectly identified a new item as having been presented; *M* = .23, *SD* = .26). An independent-samples *t*-test revealed that *A’* for participants recognizing items that were paired with cues (*M* = .85, *SD* = .15) was similar to those in the no-cue condition (*M* = .88, *SD* = .12), [*t*(60) = .79, *p* = .433, *d* = .20, BF_01_ = 2.97], thus, item recognition was similar regardless of whether or not there was a cue. Additionally, to examine differences in hit rates as a function of cue,^4^ a paired-samples *t*-test revealed that the hit rate for You items (*M* = .88, *SD* = .16) was similar to the hit rate of Friend items (*M* = .82, *SD* = .16), [*t*(29) = 1.78, *p* = .086, *d* = .32, BF_01_ = 1.28].

To determine if recognition (as measured by hits) was sensitive to participants’ importance ratings, a Gamma correlation was computed across participants. Results revealed that, overall, participants’ hits were sensitive to importance (γ = .19, *p* < .001). Additionally, we computed Gamma correlations at the participant level for each cue (You and Friend items), and these correlations (You: *M* = .17, *SD* = .67; Friend: *M* = .34, *SD* = .47) served as the dependent variable in a paired-samples *t*-test. However, results revealed that participants’ hits were similarly sensitive to importance for the items they were responsible for remembering and for items their friend was responsible for remembering [*t*(10) = .54, *p* = .603, *d* = .16, BF_01_ = 2.97].

### Confidence

To investigate differences in participants’ confidence between conditions, an independent-samples *t*-test revealed that participants in the cued condition (*M* = 78.66, *SD* = 15.10) were similarly confident as participants in the no-cue condition (*M* = 85.35, *SD* = 15.08), [*t*(59) = 1.73, *p* = .089, *d* = .44, BF_01_ = 1.10]. Furthermore, to determine if participants’ confidence differed as a function of cue, a paired-samples *t*-test revealed that participants were similarly confident for You items (*M* = 89.67, *SD* = 13.44) as Friend items (*M* = 89.11, *SD* = 21.69), [*t*(29) = .14, *p* = .887, *d* = .03, BF_01_ = 5.10].

### Strategy use

Finally, to examine differences in effective strategy use, a paired-samples *t*-test revealed that participants reported using more effective encoding strategies for You items (*M* = .24, *SD* = .24) than Friend items (*M* = .14, *SD* = .21), [*t*(29) = 2.52, *p* = .017, *d* = .46, BF_10_ = 2.83]. Thus, participants’ reported effective-strategy use replicated Experiment 1, such that participants reported using more effective strategies for You than Friend items.

## Discussion

In Experiment 2, we again examined the effects of the You and Friend cues on recall performance and sensitivity to importance by adding a control condition where words were presented without any cues and having participants provide their own importance ratings for each item. Also, rather than a surprise cue-pairing test after recall (Experiment 1), participants completed a surprise recognition test. Results revealed similar recall and recognition performance in each group and participants were similarly sensitive to item importance regardless of whether cues were present in the study phase. Thus, participants demonstrated enhanced recall and recognition for important information, consistent with engaging in responsible remembering.

In the cued condition, results generally replicated Experiment 1, such that You items were recalled better than Friend items. Thus, as evidenced by directed forgetting tasks, forgetting some items can enhance memory for target items (e.g., Bjork & Bjork, 1996; Friedman & Castel, 2011; for reviews, see Basden & Basden, 1998; Bjork, 1998; MacLeod, 1998), and when presented with to-be-remembered information, it may be beneficial to prioritize recall for the most important items or items with the biggest consequences if forgotten. However, sensitivity to importance did not differ as a function of cue, contrary to Experiment 1. Because they occurred after both tests, participants’ importance ratings may have been influenced by recall and recognition.

## Experiment 3

In Experiment 1, recall was more sensitive to item importance for You items than Friend items, but this was based on normed importance ratings. In Experiment 2, recall was sensitive to participant-level importance ratings (made after the task) regardless of the cue. In Experiment 3, we investigated if making importance ratings during encoding rather than after the task affected recall for Friend and You items or sensitivity to importance. We expected that making ratings during encoding may bias remembering and lead to reactivity (cf. Double, Birney, & Walker, 2018; Mitchum, Kelley, & Fox, 2016; Soderstrom, Clark, Halamish, & Bjork, 2015; Spellman & Bjork, 1992), whereby making judgments impacts later memory. Specifically, responsible rememberers should better remember items judged as important to remember when making the judgments during encoding rather than after the task.

## Method

### Participants

After exclusions, participants were 85 undergraduate students (age: *M* = 20.44 years, *SD* = 1.87) recruited from the University of California Los Angeles Human Subjects Pool. Participants were tested online and received course credit for their participation. Participants were excluded from analysis if they admitted to cheating (e.g., writing down answers) in a post-task questionnaire (they were told they would still receive credit if they cheated). This exclusion process resulted in two exclusions. A sensitivity analysis indicated that for a repeated-measures, between-subjects ANOVA with two groups (condition: ratings during encoding, after task) and two measurements (cue: Friend, You), with a low correlation between repeated measures (recall for Friend and You items, *r* = .08), assuming alpha = .05, power = .80, the smallest effect size the design could reliably detect is η2 = .05.

### Materials and procedure

The task in Experiment 3 was similar to the task in Experiment 2 but all items were paired with cues (as in Experiment 1). Furthermore, participants either made importance ratings at the end of the task (as in Experiment 2; *n* = 43) or made importance ratings during encoding (*n* = 42). In the latter condition, after each word was preceded by a 1-s fixation cross and appeared on the screen for 3 s, followed by the cue for an additional 2 s, participants rated the importance of remembering the item (on a scale from 0 to 100, with 0 being not important and 100 being very important). Participants were given as much time as needed to provide their ratings.

## Results

### Recall

**Fig. 4:**
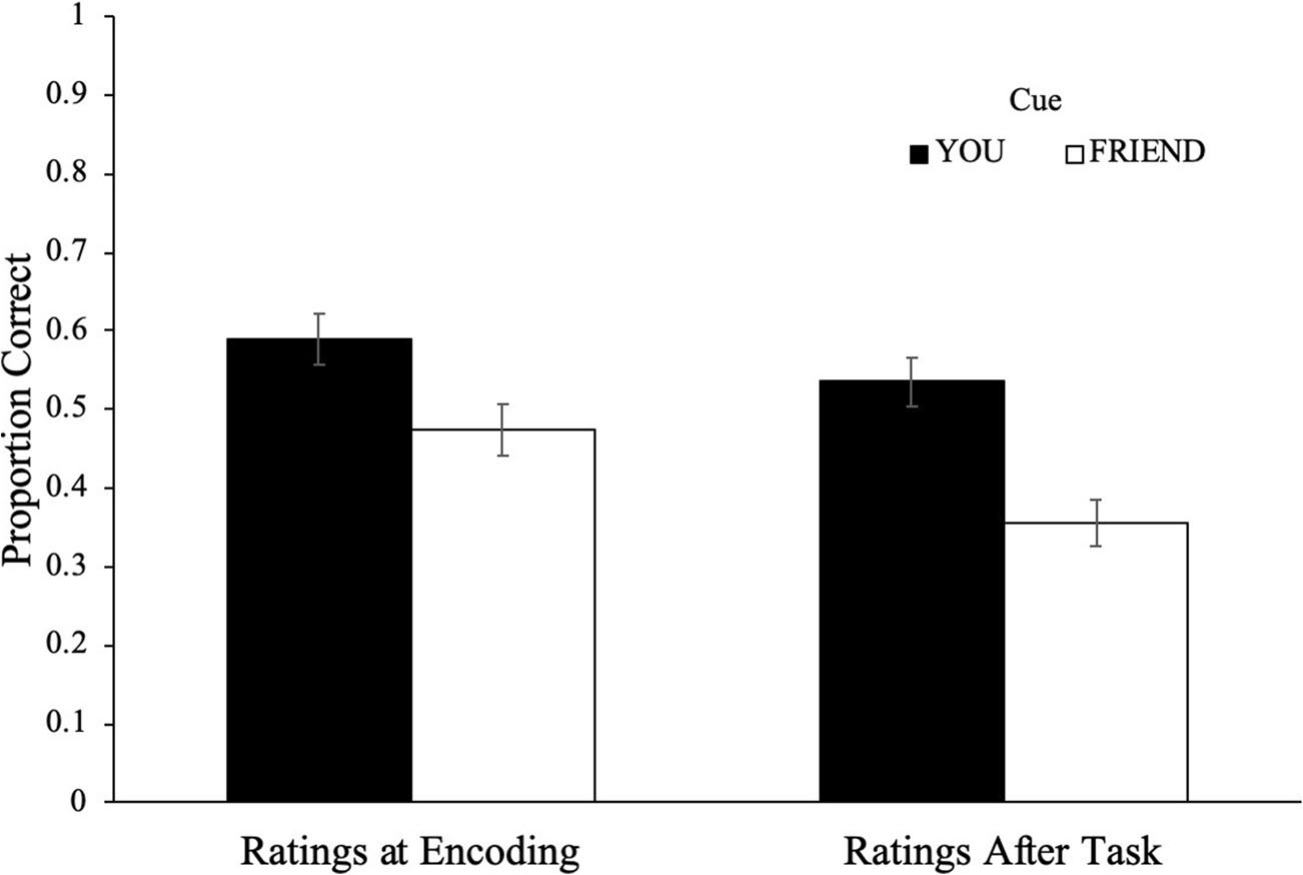
Recall performance as a function of cue and when importance ratings were made in Experiment 3. Error bars reflect the standard error of the mean

Recall performance as a function of cue and when the importance ratings were made is shown in Fig. 4. To examine differences in the proportion of words recalled, a 2 (cue: Friend, You) × 2 (condition: ratings during encoding, after task) repeated-measures ANOVA revealed a main effect of cue [*F*(1, 83) = 22.58, *p* < .001, η2 = .21, BF_10_ > 100], such that participants recalled more You items (*M* = .56, *SD* = .20) than Friend items (*M* = .41, *SD* = .22). Additionally, results revealed a main effect of condition [*F*(1, 83) = 7.05, *p* = .010, η2 = .08, BF_10_ = 2.14], such that participants making importance ratings during encoding (*M* = .53, *SD* = .16) recalled more words than participants making importance ratings after the task (*M* = .45, *SD* = .14) but cue did not interact with condition [*F*(1, 83) = 1.10, *p* = .297, η2 = .01, BF_10_ > 100].

### Output order

To elucidate how participants organized retrieval, a Gamma correlation between the output position of each correct item and the corresponding cue (You coded as 1, Friend coded as 0) was computed across participants. Results revealed that, overall, participants recalled You items before Friend items (γ = -.14, *p* = .001). At the participant level, Gamma correlations for each condition (ratings after encoding: *M* = -.23, *SD* = .52; during encoding: *M* = -.09, *SD* = .45) served as the dependent variable in an independent-samples *t*-test. However, results revealed similar organization of recall between You and Friend items according to when importance ratings were made [*t*(79) = 1.29, *p* = .202, *d* = .29, BF_01_ = 2.11].

### Importance

**Fig. 5:**
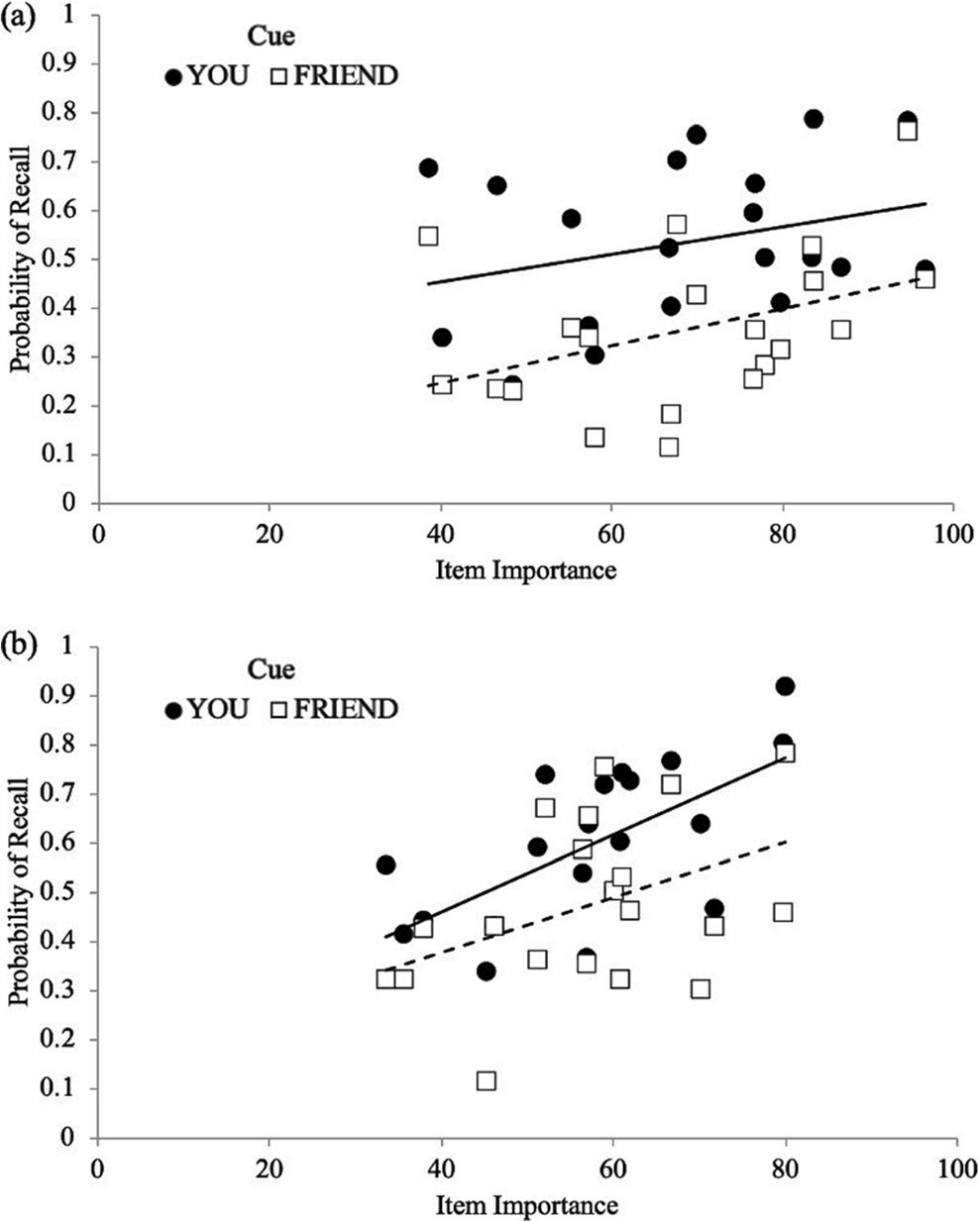
Probability of recall as a function of cue and item importance when making importance ratings at the end of the task **a** and during encoding **b** with regression lines in Experiment 3

To investigate participants’ recall as a function of item importance, a Gamma correlation between recall accuracy and item importance was computed across participants, and overall participants’ recall was sensitive to importance (γ = .20, *p* < .001). At the participant level, a 2 (cue: Friend, You) × 2 (condition: ratings during encoding, after task) repeated-measures ANOVA on recall sensitivity to importance as measured by Gammas did not reveal a main effect of condition [*F*(1, 73) = .02, *p* = .883, η2 < .01, BF_01_ = 3.79], such that participants making importance ratings during encoding (*M* = .25, *SD* = .43) were similarly sensitive to importance as participants making importance ratings after the task (*M* = .26, *SD* = .32). Additionally, results did not reveal a main effect of cue [*F*(1, 73) = .39, *p* = .534, η2 = .01, BF_01_ = 4.75], such that participants were similarly sensitive to importance for You items (*M* = .22, *SD* = .52) as Friend items (*M* = .17, *SD* = .55; see Fig. 5). Moreover, cue did not interact with condition [*F*(1, 73) = .34, *p* = .561, η2 = .01, BF_01_ = 78.07].

### Recognition

To determine whether there were differences in participants’ ability to distinguish between studied and novel items, we again calculated *A’* for each participant using hit rates (*M* = .90, *SD* = .10) and false-alarm rates (*M* = .14, *SD* = .18). To examine group differences in *A’*, an independent-samples *t*-test revealed that *A’* for participants making ratings during encoding (*M* = .94, *SD* = .12) was similar to those making ratings after the task (*M* = .91, *SD* = .08), [*t*(83) = 1.47, *p* = .147, *d* = .32, BF_01_ = 1.74].

Furthermore, to examine differences in hit rates as a function of cue, a 2 (cue: Friend, You) × 2 (condition: ratings during encoding, after task) repeated-measures ANOVA revealed a main effect of cue [*F*(1, 83) = 8.33, *p* = .005, η2 = .09, BF_10_ = 6.77], such that participants had higher hit rates for You items (*M* = .92, *SD* = .11) than Friend items (*M* = .88, *SD* = .12). Additionally, results revealed a main effect of condition [*F*(1, 83) = 13.75, *p* < .001, η2 = .14, BF_10_ = 61.07], such that participants making importance ratings during encoding (*M* = .94, *SD* = .08) had higher hit rates than participants making importance ratings after the task (*M* = .87, *SD* = .10), again suggesting reactivity as a result of making importance ratings. However, cue did not interact with condition [*F*(1, 83) = 2.92, *p* = .091, η2 = .03, BF_01_ > 100].

To investigate if recognition (as measured by hits) was sensitive to participants’ importance ratings, a Gamma correlation was computed across participants and overall, participants’ hits were sensitive to importance (γ = .17, *p* = .003). Additionally, a 2 (cue: Friend, You) × 2 (condition: ratings during encoding, after task) repeated-measures ANOVA on Gamma correlations at the participant level for each cue did not reveal a main effect of cue [*F*(1, 23) = 1.10, *p* = .305, η2 = .05, BF_01_ = 1.92], such that participants’ hits were similarly sensitive to importance for the items they were responsible for remembering (*M* = .05, *SD* = .65) and for items their friend was responsible for remembering (*M* = .24, *SD* = .64). Additionally, results did not reveal a main effect of condition [*F*(1, 23) = 3.23, *p* = .086, η2 = .12, BF_01_ = 1.11], such that participants making importance ratings during encoding were similarly sensitive to importance as participants making importance ratings after the task in terms of their hits. Also, cue did not interact with condition [*F*(1, 23) = .13, *p* = .719, η2 = .01, BF_01_ = 4.92].

### Confidence

To determine if participants’ confidence on the recognition test differed as a function of cue and time making importance ratings, a 2 (cue: Friend, You) × 2 (condition: ratings during encoding, after task) repeated-measures ANOVA did not reveal a main effect of condition [*F*(1, 83) = .57, *p* = .452, η2 = .01, BF_01_ = 2.11], such that participants making ratings during encoding (*M* = 88.31, *SD* = 15.85) were similarly confident as participants making ratings after encoding (*M* = 83.87, *SD* = 9.97). Additionally, results did not reveal a main effect of cue [*F*(1, 83) = 3.08, *p* = .083, η2 = .04, BF_01_ = 1.51], such that participants were similarly confident in You items (*M* = 92.32, *SD* = 12.91) as Friend items (*M* = 90.68, *SD* = 13.15). Moreover, cue did not interact with condition [*F*(1, 83) < .01, *p* = .970, η2 < .01, BF_01_ = 12.88].

### Strategy use

Finally, to examine differences in effective strategy use, a paired-samples *t*-test revealed that participants reported using more effective encoding strategies for You items (*M* = .38 *SD* = .32) than Friend items (*M* = .24, *SD* = .30), [*t*(84) = 4.75, *p* < .001, *d* = .52, BF_10_ > 100]. Thus, participants’ reported effective-strategy use generally replicated Experiments 1 and 2, such that participants reported using more effective strategies for items they were responsible for remembering compared with items their friend was responsible for remembering.

## Discussion

In Experiment 3, we investigated how the timing of evaluating each item’s importance affects sensitivity to item importance in recall overall, and between cues (items participants were responsible for remembering compared with items their “friend” was responsible for remembering). Consistent with Experiments 1 and 2, participants recalled more You than Friend items. Additionally, participants were sensitive to item importance overall but not as a function of cue or when participants made importance ratings. Thus, in both Experiments 2 and 3, sensitivity to importance did not differ whether the participant or their hypothetical friend was responsible for remembering each item, potentially the result of participants making their own importance ratings rather than using normed ratings. Specifically, judging an item’s importance trumped the cue indicating who was responsible for remembering it. Furthermore, judging an item’s importance during encoding lead to enhanced recall performance compared with participants making importance ratings after the task (but there were no differences in recognition), suggestive of reactivity as a result of making importance judgments (see Double et al., 2018; Mitchum et al., 2016; Soderstrom et al., 2015; Spellman & Bjork, 1992).

## General discussion

Although it may seem counterproductive, forgetting is a critical function of memory (Storm, 2011). Specifically, forgetting may be useful in situations where memory is outdated or no longer goal-relevant. While our inclination is often to try to remember as much information as we can, the notion of responsible forgetting involves overcoming this habitual behavior to focus on remembering important information at the expense of goal-irrelevant information. As a result, people may strategically rehearse and remember important information while forgetting or inhibiting unimportant information to reduce competition for goal-relevant information (cf. Anderson et al., 1994; Bjork et al., 1998). Thus, situations in which forgetting serves a useful function and aids in the recall of important information exemplify the need for responsible forgetting.

Previous research has indicated that if participants have a reliable platform with which to offload to-be-remembered information, this offloading can facilitate memory for other to-be-remembered information (Risko & Dunn, 2015; Risko & Gilbert, 2016; Sparrow et al., 2011; Storm & Stone, 2014). While *responsible remembering* (see Murphy & Castel, 2020) pertains primarily to remembering information with negative consequences if forgotten, forgetting or offloading unimportant information or items that do not need to be remembered may be a sign of *responsible forgetting*. Specifically, responsible forgetting encompasses the forgetting or offloading of less important, goal-irrelevant information to facilitate the retrieval of important, goal-relevant information by reducing competition for target information. Thus, by reducing interference for target information (by writing down or saving other to-be-remembered information), memory can be enhanced.

In the current study, participants were presented with a list of words and participants best remembered items that they were responsible for remembering at the expense of items that their “friend” was responsible for remembering, consistent with engaging in responsible forgetting.^5^ While typical item-method directed forgetting tasks provide explicit instruction or motivation to forget certain items, participants in the current study likely decreased encoding and offloaded items that their friend was responsible for remembering rather than actively attempting to forget that information. As such, participants’ recall fits with selective rehearsal accounts of directed forgetting (Benjamin, 2006; Bjork, 1972; MacLeod, 1975; Tan et al., 2020), such that participants used more effective encoding strategies and subsequently recalled more items they were responsible for remembering (participants also use more effective strategies when encoding valuable information; see Hennessee, Patterson, Castel, & Knowlton, 2019) compared with items their friend was responsible for remembering, exemplifying the benefits of forgetting (e.g., Bjork & Bjork, 1996; MacLeod, 1998). Specifically, participants used responsible remembering as a form of cognitive control to strategically prioritize encoding for more relevant or important items, facilitating retrieval of these items (Anderson, 2003; Anderson et al., 1994; Storm & Levy, 2012; but see MacLeod, Dodd, Sheard, Wilson, & Bibi, 2003; Lehman, McKinley-Pace, Leonard, Thompson, & Johns, 2001).

In addition to supporting selective rehearsal accounts of directed forgetting, the present results are also consistent with inhibitory accounts. Despite demonstrating elevated recall for items they were responsible for remembering, participants successfully recognized items both they and their friend were responsible for remembering, indicating that these items were not completely forgotten. Rather, these items may have been inhibited during recall. For example, we investigated how participants prioritized the retrieval of items according to who was responsible for remembering them. Results revealed that participants strategically organized retrieval by recalling You items before Friend items in each Experiment. Thus, during recall, output interference – the decreased recall probability as a function of later output position (Bäuml, 1998; Roediger III, 1974; Roediger III & Schmidt, 1980; Smith, 1971, 1974) may have reduced the accessibility of Friend items as a result of participants’ organization of retrieval, such that participants may have inhibited Friend items during recall to facilitate the retrieval of You items.

Although inhibition and selective rehearsal are not necessarily mutually exclusive in their impact on recall (see Fawcett, Lawrence, & Taylor, 2016), such that each mechanism may have contributed to enhanced memory for goal-relevant information in the current study, other cognitive mechanisms may underpin participants’ enhanced recall of items they were responsible for remembering. Specifically, elevated recall of You items may have resulted from the self-reference effect (see Symons & Johnson, 1997), whereby information relating to oneself is better remembered than information relating to someone else. Additionally, the relative distinctiveness principle suggests that information is well remembered to the extent that it is more distinct than competing information during recall (Neath, 2010), and You items may have been more distinct compared with Friend items, leading to better memory for these items.

When recalling items to bring on a camping trip, goal-irrelevant items appeared to benefit from schematic support (cf. Castel, 2005; Craik, 2002; Craik & Bosman, 1992; McGillivray & Castel, 2017), such that camping items that participants’ friend was responsible for remembering showed elevated recall compared with unassociated words that a participant’s friend was responsible for remembering. However, participants were similarly sensitive to the importance of these items compared with items they were responsible for remembering, indicating that they engaged in responsible remembering by prioritizing recall for the most important items. This exemplifies a key aspect of the functionality of memory, such that remembering to pack water or a tent (items rated as most important) is critically important for a camping trip, while forgetting a chair or cards (items rated as least important) is relatively inconsequential.

The act of offloading or writing down information from a list can influence memory (Risko & Dunn, 2015; Risko & Gilbert, 2016; Sparrow et al., 2011; Storm & Stone, 2014); however, it was previously unclear as to whether this process would enhance memory for important information. The results of the present study revealed that even if a friend remembering certain items is the medium with which participants offload information, participants still recalled important information that could have been offloaded. Thus, this behavior serves a functional benefit, such that in the case of an untrustworthy friend, critical information with consequences if forgotten may not be remembered and responsible rememberers should be tuned to remember important information even if this important information can be offloaded. Furthermore, forgetting should also be tuned to importance, such that it may be responsible to forget unimportant information and responsible rememberers should remember important information, even if it can be offloaded, to reduce potential consequences if forgotten.

In the present study, we focused on the goal-directed forgetting of items to pack for a camping trip. While this category of information showed benefits from schematic support, the results might be even stronger using a category of items where the end goal is not as apparent, such as food items on a shopping list. Additionally, a broader category (i.e., things to pack for a vacation) may result in more variability in importance ratings and potentially greater sensitivity to importance ratings. Furthermore, participants’ offloading strategies may differ in a dyad study. Specifically, rather than a hypothetical friend, a second participant or confederate (either trustworthy or untrustworthy) in a similar design could be responsible for remembering “friend” items to determine how offloading behavior differs in the presence of another person compared with an imaginary friend. Lastly, participants were tested immediately after encoding, but future work could institute a distractor task between study and recall to examine whether the observed effects are maintained after a delay (see Asfestani et al., 2020; Spaniol, Schain, & Bowen, 2014).

In sum, being able to focus on remembering information that has the greatest consequences if forgotten is a metacognitive process that we have termed *responsible remembering* (Murphy & Castel, 2020). Although responsible remembering typically deals with *remembering, forgetting* appears to be a critical function as well. In the present study, we demonstrated that the forgetting of goal-irrelevant information that is less important facilitates the retrieval of important target information. By understanding that they will not be able to remember all of the information, participants prioritized recall for the items they needed to remember while forgetting those that they do not need to remember. Thus, the present results provide novel evidence for *responsible forgetting*: forgetting that may be induced due to the selective rehearsal of important information that needs to be remembered (cf. Bjork, 1989; Geiselman & Bagheri, 1985; Weiner & Reed, 1969) or the inhibition of less important (Basden et al., 1993; Basden & Basden, 1998; Bjork, 1989; Geiselman et al., 1983; Geiselman & Bagheri, 1985; Weiner & Reed, 1969), such that an efficient rememberer can prioritize the retrieval of important information and/or items with negative consequences if forgotten. Taken together, the present work suggests that both responsible remembering and responsible forgetting play important roles in the strategic control of the remembering process.

## Appendix

**Table 1:** Items used in the unassociated words and schematic support (going camping, adapted from McGillivray & Castel, 2017) conditions in Experiment 1 as well as the critical lures used in Experiments 2 and 3. Stimuli were normed for word length, log-frequency, and concreteness using the English Lexicon Project website.

Unassociated words	Camping items	Experiment 2 & 3 Lures
Actor	Ax	Batteries
Cheek	Backpack	Camera
Chord	Boots	Candles
Circus	Cards	Cooler
Cliff	Chair	Flares
Dough	Clock	Gloves
Fever	Compass	Gun
Lesson	Cups	Honey
Nerve	Lantern	Knife
Palace	Marshmallows	Lighter
Prism	Matches	Map
Receipt	Pillow	Pants
Ribbon	Shovel	Radio
Scholar	Soap	Shirts
Sticker	Tarp	Socks
Sunset	Tent	Speaker
Thorn	Toothbrush	Spikes
Tunnel	Water	Sunscreen
Venue	Whistle	Swimwear
Vitamin	Wood	Towel

**Table 2:** Normed importance ratings for camping trip items used in Experiment 1 and the mean ratings from participants in Experiments 2 and 3.

Camping Items	Experiments 1 Normed Importance Ratings	Experiments 2 Mean Ratings	Experiments 3 Mean Ratings
Ax	58.3	71.8	54.5
Backpack	81.0	80.4	78.9
Boots	69.6	74.2	67.9
Cards	33.4	38.6	37.0
Chair	46.8	44.0	48.9
Clock	52.6	64.4	42.3
Compass	80.4	87.8	67.0
Cups	58.6	61.2	63.6
Lantern	81.0	86.4	70.5
Marshmallows	37.5	44.0	45.3
Matches	86.3	80.3	75.3
Pillow	61.9	68.2	58.7
Shove	56.3	66.5	52.0
Soap	68.7	69.6	62.2
Tarp	75.8	76.5	71.7
Tent	93.0	94.4	87.5
Toothbrush	73.8	71.9	74.4
water	96.9	93.2	88.5
whistle	55.6	50.8	51.9
wood	82.0	71.8	64.4

## Supplemental Experiment

In Experiment 1, participants generally recalled more You than Friend items, however, there were no differences in accuracy for these items on a surprise cue-pairing test. While these memory patterns may support inhibitory and selective rehearsal accounts of forgetting respectively, the cue-pairing test may have been contaminated by the previous recall test. In this supplemental experiment, we investigated if participants’ cue-pairing accuracy was affected by the recall test by having participants complete a distraction task instead of free recalling all to-be-remembered words (as in Experiment 1) before the surprise cue-pairing test. We also had participants provide importance ratings for each of the camping trip items to evaluate participants’ sensitivity to importance in Experiment 1. We expected to observe similar results as in Experiment 1 such that cue-pairing accuracy does not differ between You and Friend items.

## Method

### Participants

Participants were 60 undergraduate students (age: *M* = 20.12, *SD* = 1.42) recruited from the University of California Los Angeles Human Subjects Pool. Participants were tested online and received course credit for their participation. We collected the same number of participants as in Experiment 1 in an attempt to replicate the null finding of no cue-related differences on the cue-pairing test. Participants were excluded from analysis if they admitted to cheating (e.g., writing down answers) in a post-task questionnaire (they were told they would still receive credit if they cheated). This exclusion process resulted in no exclusions.

### Materials and Procedure

Participants were randomly assigned to one of two conditions: unassociated words (*n* = 30) or words offering schematic support (*n* = 30). The task in this experiment was similar to the task in Experiment 1 except that instead of a 1 minute recall test after the study phase, participants completed a 1 minute distraction task requiring them to rearrange the digits of several three-digit numbers in descending order (e.g., 123 would be rearranged to 321). Participants were given 3 seconds to view each three-digit number and subsequently retype the digits. Following the distraction task, participants were presented with the same surprise cue-pairing test as in Experiment 1. Finally, to norm the items offering schematic support for importance, after completing the cue-pairing test, participants that were presented with camping trip items were presented with these items again and asked to rate the importance of each item on a scale from 0 to 100 (with 0 being not important and 100 being very important). The average of these ratings for each item was used as the importance ratings in Experiment 1.

## Results and Discussion

***Cue pairing test.*** To examine differences in performance on the cue-pairing test^6^, a 2 (cue: Friend, You) x 2 (stimulus type: schematic support, unassociated) repeated-measures ANOVA did not reveal a main effect of stimulus type [*F*(1, 58) = .84, *p* = .363, η2 = .01, BF_01_ = 2.22], such that participants presented with words offering schematic support (*M* = .73, *SD* = .17) correctly paired a similar proportion of words as participants presented with unassociated words (*M* = .68, *SD* = .22). Additionally, results did not reveal a main effect of cue [*F*(1, 58) = 2.67, *p* = .107, η2 = .04, BF_01_ = 1.57], such that participants correctly paired a similar proportion of You items (*M* = .73, *SD* = .22) as Friend items (*M* = .68, *SD* = .23) with the correct cue. Also, cue did not interact with condition [*F*(1, 58) = .57, *p* = .453, η2 = .01, BF_01_ = 10.50]. Thus, similar to Experiment 1, participants’ associative memory for Friend and You items was not sensitive to cue or stimulus type.

Next, we investigated whether participants’ cue-pairing was sensitive to their importance ratings. A Gamma correlation across participants revealed that, overall, participants were not sensitive to importance in their cue-pairing (γ = .04, *p* = .547). Additionally, we computed Gamma correlations at the participant level for each cue (You and Friend items) and these correlations (You: *M* = .04, *SD* = .50; Friend: *M* = -.02, *SD* = .62) served as the dependent variable in a paired-samples *t*-test. Again, results revealed that participants’ associative memory was not sensitive to their importance ratings for the items they were responsible for remembering compared to items their friend was responsible for remembering [*t*(20) = .64, *p* = .532, *d* = .14, BF_01_ = 3.66]^7^, generally replicating Experiment 1.

***Confidence.*** To determine if participants’ confidence on the cue-pairing test differed as a function of cue and stimulus type, a 2 (cue: Friend, You) x 2 (stimulus type: schematic support, unassociated) repeated-measures ANOVA did not reveal a main effect of stimulus type [*F*(1, 58) < .01, *p* = .996, η2 < .01, BF_01_ = 2.01], such that participants presented with words offering schematic support (*M* = 76.17, *SD* = 19.16) were as confident as participants presented with unassociated words (*M* = 76.17, *SD* = 19.38). However, results revealed a main effect of cue [*F*(1, 58) = 9.49, *p* = .003, η2 = .14, BF_10_ = 10.92], such that participants were more confident in You items (*M* = 78.22, *SD* = 19.45) than Friend items (*M* = 74.14, *SD* = 20.12) but cue did not interact with condition [*F*(1, 58) < .01, *p* = .983, η2 < .01, BF_10_ = 1.54]. In sum, there were no differences of interest in the cue-pairing test between Experiment 1 and this experiment, thus, the cue-pairing test does not appear to have been contaminated by the recall test in Experiment 1.

## Appendix D

In Experiment 1, there were only 8 intrusions (5 in the schematic-support condition, 3 in the unassociated words condition) for an average of .13 intrusions per participant (*SD* = .34). In Experiment 2, there were 26 intrusions (15 in cue condition, 11 in no cue condition; *M* = .42, *SD* = 1.03). In Experiment 3, there were 33 intrusions (13 from participants making ratings after encoding, 20 from participants making ratings during encoding; *M* = .39, *SD* = .71).

## Footnotes

1 Participants selected You 51.6% of the time and Friend 48.4% of the time.

2 We had a different group of participants (*n* = 60) complete a distraction task instead of recalling all to-be-remembered words (as in Experiment 1) before the surprise cue-pairing test. Results replicated Experiment 1 and are included in Appendix C.

3 There were no differences in importance ratings between Friend and You items [*t*(29) = 1.00, *p* = .324, *d* = .18, BF_01_ = 3.25].

4 *A’* cannot be calculated as a function of cue because there cannot be false alarms for items from the study phase.

5 Despite some previous work suggesting that cognitive offloading can increase false recall (e.g., Lu, Kelly, & Risko, 2020), recall intrusions were rare in each experiment. Thus, the recall benefits of schematic support did not lead to many memory errors. See Appendix D for intrusion rates in each experiment.
